# Exploring the Anemia Ecology: A New Approach to an Old Problem

**DOI:** 10.1016/j.tjnut.2023.07.016

**Published:** 2023-09-30

**Authors:** Daniel J. Raiten, Denish Moorthy, Laura S. Hackl, Omar Dary

**Affiliations:** 1 Eunice Kennedy Shriver National Institute of Child Health and Human Development, National Institutes of Health, Bethesda, MD, United States; 2 USAID Advancing Nutrition, JSI Research and Training Institute, VA, United States; 3 Division of Nutrition and Environmental Health, Office of Maternal and Child Health and Nutrition, Bureau for Global Health, United States Agency for International Development, Washington, DC, United States

**Keywords:** anemia ecology, etiology, assessment methods, public health interventions

## Abstract

Our ability to identify anemia and all its permutations demands an approach that integrates the key elements of a complex “ecology,” which intertwines biology and mechanistic aspects of nutrients with both the health status and underlying factors—physical, economic, social, behavioral, demographic, and environmental. The complexity of anemia demands an ecologic approach that appreciates systems biology, translates sensitive and specific assessment methodologies and interventions, and ultimately improves clinical and public health outcomes. This series of technical papers on anemia by the U.S. Agency for International Development (USAID) Advancing Nutrition Anemia Task Force (ATF) is a first step in translating our ecologic approach to anemia with a view toward balancing research with its translation to effective programs, interventions, and policy. This introductory overview describes the components of our ecologic approach—linking the biology of anemia with its assessment and using the learning from that confluence to devise context-specific interventions. This introductory review briefly discusses the topics that underlie the biology and primary etiologies of anemia and presents a framework for public health assessment of anemia, leading to appropriate public health interventions. The other 3 manuscripts in the supplement provide the details of the arguments laid out in the introduction.

## Statement of the problem

Although the exact global prevalence of anemia is unknown, estimates place it between 23% [1] to 33% worldwide [2]. Reducing anemia prevalence has become an enduring target for strategies to improve global health, engaging a broad swath of the international research, policy, and program community. We understand that anemia decreases the oxygen carrying capacity of the organism, thus affecting critical aspects of human function and development; however, is that understanding enough to be able to accomplish our communal goals for reduction of this scourge? The following conceptual overview introduces the series of papers of the U.S. Agency for International Development (USAID) Advancing Nutrition Anemia Task Force (ATF) representing a continuum of thought:

1. Developing an understanding of the biology of anemia and its various etiologies
2. Applying that biology to its assessment
3. Developing and applying a framework to translate that science to inform choice about context-specific interventions to address anemia in individuals and populations.

Anemia coexists with pandemics of food/nutrition insecurity, malnutrition, and infectious and noncommunicable diseases. The manifestations of this confluence are most severe in women, infants, and children [3]. Our ability to identify anemia and all its permutations demands an approach that integrates the key elements of a complex “ecology.” Box 1 highlights critical operating assumptions about how to address anemia. (The ATF papers describe how genetic factors may influence anemia, other hematologic conditions, and iron status. The ATF working group chairs recognize that race and ethnicity are social constructs that lack a biological foundation and fail to take account of the extent of variability in the myriad nongenetic determinants of physiology [4,5]. Accordingly, the task force will not use racial and ethnic classifications for reference intervals in diagnostic evaluation or for other descriptive purposes.)

BOX 1 **Operating assumptions about how to address anemia. These are our assumptions that underpin our approach to anemia.**

Operating Assumptions

- Anemia, which is a reflection of the health of the hematological system, has multiple causal factors (i.e., an ecology existing of genetics, disease, nutrition, and the environment).
- An ecology defines the interactions between a complex system, here anemia’s interactions with internal (biology, health context) and external (nutrition, food insecurity, physical, social, and behavioral) environments.
- Our understanding of the components of the anemia ecology is critical to our efforts to identify and treat with precision at both individual and population levels.

Alt-text: BOX 1

Anemia exemplifies the challenges of the global health and nutrition enterprise (i.e., how to understand health and disease in a complex global health environment, how to assess a complex problem, and how to develop context-specific interventions to address that complexity). Fundamentally, if reducing anemia is the public health target, we need to answer critical questions, such as the following:

1. What are the contributions of various factors (genetic, physiologic, and nutritional) to the causation of anemia?
2. Among the nutritional causes of anemia, which of the following nutrients—iron, folate, vitamin B_12_, vitamin A, riboflavin, and perhaps, vitamin B_6_—are responsible, individually or in combination?
3. How do we address challenges of assessment, including:
  — How do we make a differential diagnosis about causes of anemia particularly at population levels?
  — What are appropriate approaches for measuring hemoglobin (venous and/or capillary blood samples)? [6]
  — Can/should we make judgments about the role of nutrition in the absence of relevant biomarkers of iron or other suspected nutrients and indicators of infection/inflamm-ation?

The presumption in many circles is that anemia is synonymous with iron deficiency, and the latter is commonly attributed to only low-iron intakes when infection/inflammation are equally important causes of functional iron deficiency. A common view persists that 50% of anemia is caused by iron deficiency [7]; however, recent estimates indicate that perhaps as low as 25% or 37% in preschool children and women of reproductive age, respectively, of anemia is because of iron deficiency [8].

Although all anemia is not iron deficiency and all iron deficiency is not anemia, iron deficiency remains a significant factor in our ability to comprehensively address anemia—and a significant global health concern in its own right. Despite significant strides to reduce its prevalence, iron deficiency affects ∼40% of the world’s women [9]. Although iron deficiency remains the most significant micronutrient deficiency worldwide (either because of low-iron intakes or low bioavailability or impaired metabolism because of infection/inflammation and parasitism), the conflation of iron deficiency with anemia impairs our ability to address anemia comprehensively from a public health perspective. (The ATF makes the following distinction between bioavailability and micronutrient absorption: bioavailability reflects the physiochemical properties of the micronutrient form, food matrix, the composition of the diet, and interactions with the gut environment, whereas absorption is a metabolic process that also includes the consumer’s health context as influenced by developmental stage, genetics, infections, inflammation, etc. Within the context of the overall ATF conceptual approach, bioavailability is a reflection of the external environment, and absorption is the combined influence of external and internal environments.) In addition to the challenges of making differential diagnoses about prevalent causes of anemia, the following factors also impact our ability to address iron deficiency:

1. Because of the myriad of factors that affect it, questions remain about the utility of the hemoglobin concentration, which although valuable if measured correctly, as a bioindicator of anemia, lacks the sensitivity and specificity to serve as a biomarker of iron status and thus is not an appropriate trigger for public health interventions to address iron nutrition [10].
2. Safety concerns exist about iron supplementation, particularly in the context of malaria or other infections [11].
3. Limitations exist in using and interpreting current biomarkers to distinguish between nutritional (dietary) iron deficiency and responses to infection/inflammation [12].
4. There is a lack of precision in our ability to determine the amount of remedial iron needed to improve and maintain iron status to ensure health, particularly in different disease or geographic contexts (in individuals or populati-ons).

In the absence of answers to these questions, our ability to intervene is compromised both at the individual and population level. The complexity of anemia demands an ecologic approach that appreciates systems biology, translates sensitive and specific assessment methodologies and interventions, and ultimately improves clinical and public health outcomes. Our ability to assess iron status and deficiency in individuals or populations is challenged by the inability to differentiate between a dietary insufficiency and a physiologic response to a concurrent health issue (e.g., infection and/or inflammation).

**FIGURE 1:**
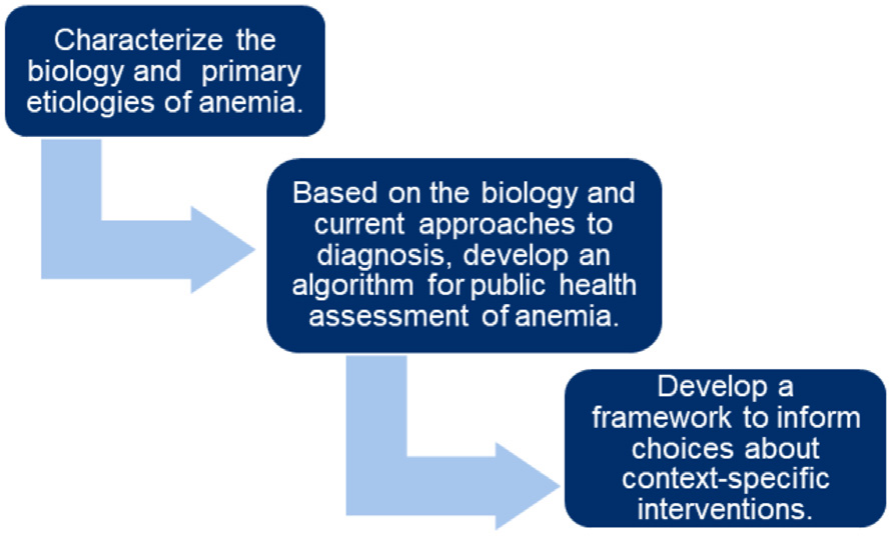
A stepwise approach to addressing anemia. Our stepwise approach to anemia includes an understanding of biology, linking biology and biological mechanisms to assessment of anemia, and finally translating biology and assessment information to action via effective interventions.

In an effort to address the range of issues described above, the USAID Advancing Nutrition ATF organized this series of papers based on the continuum of thought represented in Figure 1. The following sections provide a conceptual framework for addressing anemia used by the ATF in its deliberations. The 3 papers in this supplement are linked and are informed by each other. Brittenham et al. [13] describe the systemic and biologic underpinnings of anemia, which is connected to the review of the assessment of anemia and its causes by Williams et al. [14]. Loechl et al. [15] use the findings in the biology and assessment of anemia papers to suggest evidence-based context-specific interventions.

## Biology of anemia prevalence and primary etiologies

When considering the causes of anemia, the review by Kassebaum et al. [16] is the point of reference for determining the prevalence and primary causes of anemia globally. Although a myriad of potentially concomitant causes of anemia exists [17], for the purposes of the ATF deliberations, key elements of the anemia ecology highlighted in Figure 1 were adapted to provide focus. Specifically, we focus on the 3 core classes of etiology: genetics, health status (e.g., infection/inflammation), and nutrition, as they reflect the most common etiologies and exemplify the nature of the challenges associated with the biology, assessment, and translation to interventions confronting the global health enterprise.

### Nutritional causes of anemia

#### Iron-specific issues

Based on their analysis, iron deficiency anemia (IDA) remains the leading cause of anemia globally. Although iron deficiency was identified as the leading cause of anemia globally, the analysis of Kassebaum et al. [16] was not based on iron biomarker data (rather, residual unexplained anemia). Kassebaum et al. [16] concluded: “If we include those conditions such as hookworm, schistosomiasis, and gastrointestinal and gynecologic conditions that also manifest as IDA, its importance grows even more apparent.”

They also observed: “Despite its continued dominance, a decrease in IDA has been the primary driver of reduced global anemia burden since 1990,” and added that “the improvements have been partially offset; however, by increases in anemia owing to chronic kidney diseases, hemoglobinopathies, malaria, and schistosomiasis. Most of the increase in these latter conditions is related to population aging (for chronic kidney diseases) and population growth in endemic areas.”

These conclusions clearly highlight the following:

1. The complexity of the anemia challenge
2. The need to be able to distinguish between nutrition and physiology (functional anemia due to infection/inflammation) because the inclusive characterization of IDA used by Kassebaum et al. [16] above encompasses both nutritional and functional iron deficiency (i.e., measurable deficits in biomarkers of iron status that may be the result of nonnutritional causes).

To add to the confusion, Kassebaum et al. [16] also suggested:

“IDA in high-risk, high-prevalence populations is likely due to impaired micronutrient intake or bioavailability or absorption or mobilization. Addressing IDA by only increasing micronutrient supplementation would not be a panacea for ending anemia, however, because IDA is a final common pathway of a heterogeneous group of conditions that also includes acute or chronic hemorrhagic events and disorders of iron metabolism that may happen as a result of chronic illness or malabsorption.”

Although an important acknowledgment of the role of iron in anemia, this perspective perpetuates the perception about the predominance of: *1*) iron deficiency and *2*) conflates nutritional iron deficiency with impaired iron physiology due to a myriad of causes.

Core questions remain (which we address in detail in both the Biology [13] and Assessment [14] papers in this supplement):

1. How do the physiology and response to infection/disease blend with the specific aspects of dietary iron nutrition and bioavailability?
2. How should we represent IDA in the context of anemia, given the latter’s multifactorial causation? Can we improve our precision by moving away from its use as an umbrella term inclusive of not only nutritional iron deficiency, but also the potential role of multiple other nutrients—as well as the use of iron status to reflect multiple responses to a challenged physiology (i.e., infection, parasites, and blood loss)?

#### Other nutritional causes of anemia

Clinicians and researchers have linked multiple nutrients to anemia. A recent meta-analysis of the impact of both nutrition-specific and nutrition-sensitive interventions on hemoglobin/anemia documented an inconsistent response to specific nutrient interventions (e.g., vitamin A, vitamin C, zinc, and multiple micronutrient supplements) as opposed to a relatively consistent response to iron or iron-folic acid supplementation [18]. Whether these findings reflect a limitation in available data and/or an incomplete understanding of the anemia ecology needs to be determined.

There is clearly an association between nutritional status, broadly, and the status of specific micronutrients. The question is: which of them has the greatest public health implications, and how best should we assess and intervene? The ATF focused on iron, folate, vitamin B_12_, vitamin A, riboflavin, and vitamin B_6_ because these nutrients have the greatest public health implications based on prevalence of insufficiency. However, limiting the discussion to these nutrients does not imply that we should exclude other nutrients (micro or macro) from consideration in either the etiology or assessment of anemia, if the context suggests otherwise (e.g., local prevalence of food/nutrition insecurity and malnutrition).

## Anemia assessment

**FIGURE 2:**
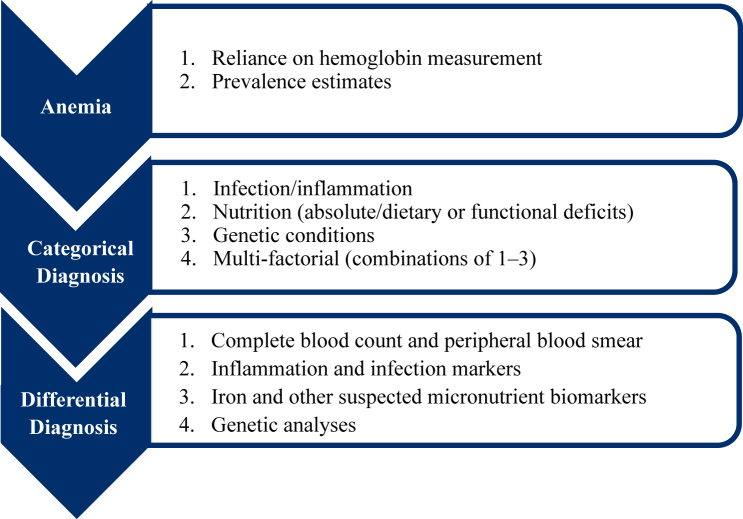
Anemia assessment process. The anemia assessment process begins by estimating the prevalence of anemia by accurate hemoglobin measurement, followed by a measurement of categorical causes, such as infection/inflammation, nutrition deficits, genetic conditions, or a combination of the 3. Within those categories, we carry out a differential diagnosis by conducting additional tests until we identify the cause (or causes).

The ATF focused on public health approaches to addressing anemia with a recognition that ultimately the target is improved health of people. Figure 2 describes an overall approach to assessment based on the biology of anemia.

### Assessment: vernacular and expectation

Elucidating the interrelationships of diet, nutritional status, health, and anemia depends on the methods and designs of observational, preclinical, and clinical studies conducted to:

1. Evaluate programs, policies, and guidance.
2. Develop and support standards of clinical care.
3. Improve individual and population health outcomes.

The value and translatability of the results of such efforts demand a vernacular that is clear in terms of sensitivity and specificity as well as expectations. The ecologic approach outlined above provides an overarching framework for anemia assessment. Methodologies for the clinical assessment of patients and the surveillance of populations should also consider an ecologic approach to address core questions about the assessment of anemia and its causes, as covered by Williams et al. [14] in this supplement.

The design of rigorous and reproducible studies to characterize the relationships of diet/nutrition and health is a critical component of anemia, prevention, care, and treatment efforts. Validated and reliable assessment tools appropriate for the questions asked and the specifics of the health and environmental contexts must address the following:

1. Nutrient exposure—bioavailable amounts of nutrients consumed
2. Nutritional status—accepted definitions for adequacy, marginal status, and deficiency
3. Nutrient function—the metabolic role(s) of specific nutrients
4. The relevant functional outcome(s).

Finally, it is critical to recognize that individuals are seldom deficient in single nutrients; multiple nutrients in metabolic systems typically function in synergy. Moreover, these factors are not globally common but vary from country to country and from community to community. These facts should inform our efforts to develop more precise assessment methodologies and study designs [19].

The ability to determine the health status of an individual (i.e., the absence or presence of disease and potential concomitants) can be difficult to achieve. Clinicians must decide not only what biomarker, but also in what biological milieu (e.g., whole blood, plasma/serum, urine, etc.), and what other factors to assess based on their potential to influence the response and/or significance of the analytic results. The Biomarkers of Nutrition for Development project [20] employed an ecologic approach to provide evidence-informed advice to researchers (basic, clinical, and surveillance), clinicians, program developers/monitors, and policy makers who rely on biomarkers to evaluate and make decisions about the role of nutrients in health.

For the purposes of their deliberations, the ATF used the following definitions:

1. Biomarkers: reflect the amount of specific nutrients interpreted within a specific biological context (i.e., health or disease).
2. Bioindicators: reflect perturbations in biological systems (e.g., electroencephalography reflects aberrations in neurophysiology, hemoglobin reflects hematologic status, C-reactive protein reflects inflammation) but not specific nutrient status (i.e., they cannot serve as a biomarker of particular nutrients).
3. Public health indicators: reflect disturbance in the external ecology (e.g., socioeconomic status, food insecurity, disability-adjusted life years, or stunting or underweight). Because researchers often use these indicators to suggest causality, this data trigger public health interventions.

Bioindicators and public health indicators may be nutritionally sensitive without being nutrient-specific. As such, practitioners cannot reliably use them to make inferences about diet/nutrition and health without supporting information [21].

Two additional key issues emerged from the Biomarkers of Nutrition for Development project’s deliberations that are relevant here:

1. We need to recognize that nutrients often interact in ways relevant to the selection and interpretation of biomarkers.
2. Nutrient utilization, status, and function can affect and be affected by key biological/physiologic systems and responses.

The reciprocal relationships between nutrition and inflammation exemplify the latter [22]. In addition, we have come to appreciate the impact of the inflammatory response on the performance and interpretation of nutritional biomarkers [12].

### Some considerations about the importance of inflammation

Inflammation represents a sequence of events with profound metabolic and nutritional implications. Our ability to address nutrition in the context of inflammation is challenged by the following:

1. The direct impact on nutritional biology (i.e., implications for all the processes involved in achieving nutritional status)
2. The impact of inflammation on assessment and interpretation of biomarkers of nutrient status.

The nature of the bidirectional relationship between nutrition and inflammation (i.e., each affects and is affected by the other) has been reviewed [22]. With the observed increasing prevalence of widening obesity, we need additional research to delineate the role of specific systemic inflammatory responses seen in autoimmune diseases from the generalized inflammation associated with obesity. The implications of inflammation, and how to account for its impact on biomarker interpretation, is particularly relevant to efforts to assess and treat anemia—and has been the focus of a series of studies conducted under the Biomarkers Reflecting Inflammation and Nutritional Determinants of Anemia (BRINDA) project [[23], [24], [25], [26], [27]]. Issues specific to the intersection of inflammation with the biology and assessment are covered in detail in the relevant sections of the ATF papers in this supplement.

## Anemia—informing context-specific choices about interventions

The article by Loechl et al. [15] in this supplement explores how best to synthesize the key aspects of anemia biology and assessment to inform choice about context-specific interventions. Recognizing the importance of these considerations to clinical care, the primary focus for the ATF was on interventions of public health relevance (i.e., those deliverable at-scale and in a sustainable manner to large population groups). Given the multifactorial nature of anemia, we consider the clinical context when exploring intervention priorities. Some causes of anemia (e.g., inherited blood disorders) are managed at health facilities by expert personnel, and the efficiency of the system of referral and follow-up varies by geographic and resource contexts. A framework is presented for prioritization that expands on the ecologic underpinning of the ATF’s approach to allow consideration of a broader perspective of sustainability, with a focus on relevant social, cultural, and environmental factors. Although individual interventions are presented, either independently or in combination, we acknowledge the importance of these broader considerations.

This series of technical papers on anemia by the ATF is a first step in translating our ecologic approach to anemia with a view toward balancing research with its translation to effective programs, interventions, and policy.

## Author contributions

The authors’ responsibilities were as follows – DJR, OD, LH, DM: designed the review; DJR: wrote the paper; DM: has primary responsibility for final content; and all authors: read and approved the final manuscript.

## Conflicts of interest

The authors report no conflicts of interest.

## Funding

This article is published as part of a supplement sponsored by JSI Research & Training Institute, Inc. This manuscript was developed by the USAID Advancing Nutrition Anemia Task Force (ATF), which is supported by the U.S. Agency for International Development. The review manuscript was prepared under the terms of contract 7200AA18C00070 awarded to JSI Research & Training Institute, Inc. (JSI). USAID staff participated in the ATF meetings and working groups in their capacity as anemia experts and served as coauthors of the review papers. The contents of this manuscript are those of the authors and do not necessarily represent the official position of the United States Agency for International Development, the National Institutes of Health, or the U.S. government.
